# Accelerated cortical thinning precedes and predicts conversion to psychosis: The NAPLS3 longitudinal study of youth at clinical high-risk

**DOI:** 10.1038/s41380-022-01870-7

**Published:** 2022-11-25

**Authors:** Meghan A. Collins, Jie Lisa Ji, Yoonho Chung, Cole A. Lympus, Yvette Afriyie-Agyemang, Jean M. Addington, Bradley G. Goodyear, Carrie E. Bearden, Kristin S. Cadenhead, Heline Mirzakhanian, Ming T. Tsuang, Barbara A. Cornblatt, Ricardo E. Carrión, Matcheri Keshavan, Wiliam S. Stone, Daniel H. Mathalon, Diana O. Perkins, Elaine F. Walker, Scott W. Woods, Albert R. Powers, Alan Anticevic, Tyrone D. Cannon

**Affiliations:** 1 Department of Psychology, Yale University, New Haven, CT USA; 2 Department of Psychiatry, Yale University School of Medicine, New Haven, CT USA; 3 Interdepartmental Neuroscience Program, Yale University School of Medicine, New Haven, CT USA; 4 Department of Psychiatry, McLean Hospital and Harvard Medical School, Belmont, MA USA; 5 Institute for Technology in Psychiatry, McLean Hospital, Belmont, MA USA; 6 Center for Neuroscience, University of Pittsburgh, Pittsburgh, PA USA; 7 Department of Psychiatry, Hotchkiss Brain Institute, University of Calgary, Calgary, AB Canada; 8 Department of Radiology, Hotchkiss Brain Institute, University of Calgary, Calgary, AB Canada; 9 Departments of Psychiatry and Biobehavioral Sciences and Psychology, Semel Institute for Neuroscience and Human Behavior, UCLA, Los Angeles, CA USA; 10 Department of Psychiatry, UCSD, San Diego, CA USA; 11 Institute of Genomic Medicine, UCSD, La Jolla, CA USA; 12 Department of Psychiatry, Zucker Hillside Hospital, Long Island, NY USA; 13 Division of Psychiatry Research, The Zucker Hillside Hospital, Glen Oaks, NY USA; 14 Institute of Behavioral Science, The Feinstein Institutes for Medical Research, Manhasset, NY USA; 15 Department of Psychiatry, The Donald and Barbara Zucker School of Medicine at Hofstra/Northwell, Hempstead, NY USA; 16 Department of Psychiatry, Harvard Medical School at Beth Israel Deaconess Medical Center and Massachusetts General Hospital, Boston, MA USA; 17 Department of Psychiatry, UCSF, and SFVA Medical Center, San Francisco, CA USA; 18 Department of Psychiatry, University of North Carolina, Chapel Hill, NC USA; 19 Departments of Psychology and Psychiatry, Emory University, Atlanta, GA USA

**Keywords:** Neuroscience, Predictive markers, Schizophrenia, Diagnostic markers, Psychology

## Abstract

Progressive grey matter loss has been demonstrated among clinical high-risk (CHR) individuals who convert to psychosis, but it is unknown whether these changes occur prior to psychosis onset. Identifying illness-related neurobiological mechanisms that occur prior to conversion is essential for targeted early intervention. Among participants in the third wave of the North American Prodrome Longitudinal Study (NAPLS3), this report investigated if steeper cortical thinning was observable prior to psychosis onset among CHR individuals who ultimately converted (CHR-C) and assessed the shortest possible time interval in which rates of cortical thinning differ between CHR-C, CHR non-converters (CHR-NC), and health controls (HC). 338 CHR-NC, 42 CHR-C, and 62 HC participants (age 19.3±4.2, 44.8% female, 52.5% racial/ethnic minority) completed up to 5 MRI scans across 8 months. Accelerated thinning among CHR-C compared to CHR-NC and HC was observed in multiple prefrontal, temporal, and parietal cortical regions. CHR-NC also exhibited accelerated cortical thinning compared to HC in several of these areas. Greater percent decrease in cortical thickness was observed among CHR-C compared to other groups across 2.9±1.8 months, on average, in several cortical areas. ROC analyses discriminating CHR-C from CHR-NC by percent thickness change in a left hemisphere region of interest, scanner, age, age^2^, and sex had an AUC of 0.74, with model predictive power driven primarily by percent thickness change. Findings indicate that accelerated cortical thinning precedes psychosis onset and differentiates CHR-C from CHR-NC and HC across short time intervals. Mechanisms underlying cortical thinning may provide novel treatment targets prior to psychosis onset.

## Introduction

Identifying mechanisms that underlie psychosis onset is essential for enhanced risk identification and early intervention. Prior work has demonstrated accelerated cortical thinning among clinical high-risk (CHR) individuals who convert to psychosis [1–6], suggesting that disruptions in mechanisms underlying neuromaturation across adolescence and young adulthood may contribute to psychosis onset [7–9]. Most notably, among a large sample of CHR individuals in the second phase of the North American Prodrome Longitudinal Study (NAPLS2), an accelerated rate of thinning in right superior frontal, middle frontal, and medial orbitofrontal regions was observed among participants who converted to psychosis, across approximately one year between pre- and post-onset [1].

Disrupted synaptic activity and neuronal connectivity are posited as underlying mechanisms of psychosis [9, 10] that likely have a progressive course that intensifies as illness develops. These mechanisms may be indirectly observable through steeper rates of cortical thinning pre-conversion (e.g., possibly through dysregulated synaptic plasticity and/or complement system activation resulting in overabundant synaptic pruning) [7, 8, 10]. However, prior investigations have not assessed changes in grey matter prior to psychosis onset, and thus cannot definitively state whether grey matter reduction precedes or follows the transition to psychosis.

Determining whether grey matter changes occur prior to conversion is essential for elucidating illness-related neurobiological processes potentially targetable by preventative interventions. Prophylactic administration of antipsychotic medications to CHR individuals is not recommended due to serious adverse side effects [11], particularly given that only 15–25% of CHR individuals will develop a psychotic disorder within two years from ascertainment [12–14]. Validating progressive cortical thinning as a predictive biomarker of psychosis may facilitate the development of novel treatments that could potentially underlie this phenomenon, such as inadequate NMDA-dependent synaptic plasticity [7, 15–18] and/or excessive neuroinflammation (microglial activation) [19–22]. It is also critical to determine the minimum possible interval at which differential rates of grey matter change are observable between CHR individuals who do and do not ultimately develop a psychotic disorder, in order to improve risk prediction and identify candidates for targeted interventions [23].

In its third phase, NAPLS recruited new cohorts of CHR and healthy controls (HC) for an intensive longitudinal biomarker follow-up study. Participants completed up to five neuroimaging and clinical assessments across approximately 8 months. Our objectives were: (1) to determine if longitudinal trajectories of grey matter change differed among CHR participants who ultimately converted to psychosis (CHR-C), those who did not convert (CHR-NC), and HC; (2) to further evaluate brain regions in which cortical thinning rates differed by group; and (3) to determine the shortest possible interval in which CHR-C cases could be differentiated from CHR-NC and HC, at the individual subject level.

## Materials/subjects and methods

NAPLS3 is a multisite cohort study conducted between 2015 and 2020 by 9 programs focusing on CHR youth. Participants included in this report completed between 1 and 5 structural magnetic resonance imaging (MRI) scans (targeted for baseline and 2, 4, 6, and 8-month follow-up). If a participant converted to psychosis during the study, a full clinical and biomarker assessment was attempted at that time.

### Participants

NAPLS3 participants included 560 CHR and 96 HC participants enroled for longitudinal biomarker follow-up, aged between 12 and 30 at baseline. Institutional Review Board committees at each site approved study protocols and all participants provided informed consent.

CHR participants were help-seeking and self-referred or were referred through medical providers, educators, and/or social service agencies. CHR cases met the Criteria of Psychosis-Risk Syndromes (COPS) based on the Structured Interview for Psychosis-risk Syndromes [24]. Exclusion criteria were: a current or lifetime history of a psychotic disorder, central nervous system disorder, psychosis-risk symptoms that were clearly caused by another Axis 1 disorder, or IQ less than 70. In addition, HC participants could not have a family history of psychosis among first degree relatives, could not have a Cluster A personality disorder diagnosis, and could not be using psychotropic medication at the time of study. The NAPLS3 study design is described in detail elsewhere [25].

After excluding participants with incomplete data and who did not pass MRI quality control standards, 62 HC and 380 CHR participants (42 CHR-C, 338 CHR-NC) were included in the present analyses. Key participant demographics are provided in Table 1. Demographics were similar among participants who met inclusion criteria and who completed neuroimaging but were excluded (Table S3), with several differences (Table S4).

**Table 1:** Participant characteristics by clinical group.

Characteristic	HC N = 62	CHR-NC N = 338	CHR-C N = 42	Statistic
Sex (at birth)				
Male	31 (50%)	188 (56%)	25 (60%)	χ^2=^1.0, ns
Female	31 (50%)	150 (44%)	17 (40%)	
Race/Ethnicity^a^				
Non-Hispanic white	26 (42%)	163 (48%)	21 (50%)	χ^2=^0.9, ns
Racial/Ethnic minority	36 (58%)	175 (52%)	21 (50%)	
* N* Taking Antipsychotic Meds^b^	N/A	93 (28%)	22 (52%)	χ^2=^9.1, **
* N* No Antipsychotic Meds	N/A	245 (72%)	20 (48%)	
Mean (SD)				
Age (first scan)	19.3 (4.3)	19.2 (4.1)	19.7 (4.5)	F = 0.24, ns
Number of Scans	3.7 (1.2)	2.7 (1.5)	2.7 (1.2)	F = 13.2, ***
Income^c^	3.9 (1.9)	4.2 (1.8)	4.3 (1.9)	F = 0.7, ns
Total SOPS^d^ Positive Symptoms (Baseline)	1.1 (1.7)	12.8 (3.4)	14.2 (3.6)	F = 363.8, ***
Total SOPS Negative Symptoms (Baseline)	1.4 (2.1)	12.1 (6.1)	15.0 (6.9)	F = 100.6, ***
Global Assessment of Functioning (Baseline)	88.3 (7.5)	50.9 (11.7)	44.9 (11.6)	F = 311.1, ***

Healthy control (HC), clinical high-risk non-converter (CHR-NC) and converter (CHR-C) participants were compared on baseline demographic and clinical indicators. P-value terms: ns >0.05; *<0.05; **<0.01; ***<0.001.

^a^Participants self-identified their racial background from one of ten categories: First Nations, East Asian, Southeast Asian, South Asian, Black, Central/South American, West/Central Asia and Middle East, White, Native Hawaiian or Pacific Islander, Interracial. Participants self-identified as non-Hispanic or Hispanic. In this report, racial/ethnic majority refers to non-Hispanic white individuals, whereas racial/ethnic minority refers to Hispanic and/or non-white individuals.

^b^Table reflects the number of participants taking antipsychotic medication at the time of at least one neuroimaging visit.

^c^Participants self-identified their household income before taxes on a 1–7 scale: 1=less than $10,000, 2 = $10,000 to $19,999, 3 = $20,000 to $39,999, 4 = $40,000 to $59,999, 5 = $60,000 to $99,999, 6 = $100,000 and above, and 7=Don’t know or refused to answer. Participants who did not report their income (N = 68) were excluded from mean/SD calculations.

^d^The Scale of Psychosis-risk Symptoms (SOPS) is a 19-item scale embedded within the SIPS [24] that assesses four domains of attenuated psychotic symptoms: Positive, Negative, Disorganization, and General Symptoms.

### Procedures

#### MRI quality control

Each T1-weighted image underwent rigorous visual quality control (QC) consisting of independent ratings by 2–3 highly trained investigators to assess for artifacts due to motion, skull strip errors, segmentation failures, white and pial surface misplacements, and/or topological defects (Table S1, S2).

#### MRI inter-scanner reliability

NAPLS3 included a separate travelling participants study to assess the between-site and test-retest reliability of MR scans using intraclass correlations (ICC). Each site recruited one healthy subject (5 male, 4 female) who was scanned on two successive days at every site. ICCs were calculated within and across scanners. This process indicated that 2 scanners were considerably less reliable than the other 7 scanners. The average ICC estimate across Desikan Killiany atlas [26] cortical parcels and subcortical/ventricular volumes increased from 0.72 when all scanners were included to 0.88 when these 2 scanners were removed. Due to substantial differences in reliability and differences in data quality observed through visual QC, both of which result in reduced power to detect change over time and between subjects, scans from these scanners were excluded. Scans collected on a third scanner (for which a small number of subjects were evaluated before hardware was upgraded) were also excluded due to concerns with reliability and poor data quality observed during visual QC (Table S5, S6, Fig. S1).

#### MRI processing

T1-weighted structural MR images were processed using the Human Connectome Project’s Minimal Preprocessing Pipelines [27] with the open-source Quantitative Neuroimaging Environment & Toolbox (QuNex, qunex.yale.edu). Automatic whole-brain segmentation and surface-based cortical reconstruction was run with FreeSurfer v6.0 [28, 29]. The FreeSurfer longitudinal pipeline [30] was applied by registering each T1 image to an unbiased within-subject template using robust, inverse consistent registration [31]. Thickness maps were resampled from native subject space to a common space (fsaverage5) for group-level analyses. A 10 mm full-width half-maximum Gaussian smoothing kernel was applied to increase the signal-to-noise ratio and reduce the effects of inaccuracies in spatial registration while preserving true regional effects [32, 33].

### Statistical analyses

Discovery analyses were performed to determine if rates of cortical thinning differ among CHR-C, CHR-NC, and HC. The FreeSurfer MATLAB toolbox [34, 35] was used to build spatiotemporal linear mixed effects (LME) models at the vertex-level to assess the interaction effect of diagnostic group-by-time on longitudinal cortical thickness. Vertex-wise models included time from first scan, group (HC, CHR-NC, CHR-C), group-by-time interaction, age, age^2^, sex, and scanner as fixed effect predictors and a random subject-specific intercept, after it was determined that model fit was not improved by including a random slope term. 95% of participants who met inclusion criteria for this report completed all scans within 12 months from baseline. Thus, to avoid bias in the model due to a right skew in the data, 23 scans collected more than 12 months from baseline were excluded from analyses.

Group-by-time F-test maps were thresholded at p < 0.01 (uncorrected) and clustered using the HCP [36] workbench command *metric-find-clusters* to retain regions with an area of at least 100 mm^2^. To empirically assess cluster significance, 1000 bootstrap replicates were created by permuting the data by group labels only (preserving longitudinal scans within subject). For each cluster, T-statistics were calculated for each group contrast, applying FDR correction across all clusters.

To determine the shortest time interval at which differential changes in cortical thickness were observable among participants who ultimately converted to psychosis, the percent change (PC) in cortical thickness between first and second scan (PC_scan2_) was calculated for participants who completed at least two scans. PCscan_2_ was assessed separately in a region comprised of all left hemisphere clusters (left ROI) and right hemisphere clusters (right ROI) obtained through LME analyses. PCscan_2_ was calculated as: (T_2_-T_1_/T_1_)/time x 100, where T_2_ = thickness at second scan, T_1_ = thickness at first scan, and time = the number of months from first to second scan. Linear regression models assessed relationships between PC_scan2_ and final clinical group status, including age at first scan, age^2^, sex, and scanner as covariates, applying FDR correction across ROIs. PC analyses were also performed between first and last scan (PC_Final_) to assess whether group differences become more pronounced across time.

Subsequently, logistic regression analyses were conducted to determine if PC_scan2_ and PC_Final_ in the left ROI could predict whether a CHR participant ultimately converted to psychosis at the individual level, including, age, age^2^, sex, and scanner as covariates. A receiver operating characteristic (ROC) curve was then calculated based on model predictions, and the sensitivity and specificity of the model was assessed by calculating area under the ROC curve (AUC). Nonparametric bootstrapping with 10,000 replications was conducted using the *boot* package [37] in R to determine a 95% confidence interval for the AUC estimate.

For comparison with prior work in NAPLS2 [1], supplementary LME analyses were performed for volumetric measures, including intracranial volume as an additional covariate (Table S10). Follow-up analyses were conducted to examine the effects of antipsychotic medications on all LME and percent change statistical tests (Tables S7–S9, Fig. S5). Additionally, PC_scan2_ and PC_Final_ effect sizes were calculated in three regions (right superior frontal, middle frontal, and medial orbitofrontal cortex) in which CHR-C demonstrated accelerated cortical thinning relative to CHR-NC and HC in NAPLS2 [1] (Cohen’s d). Effect sizes in the left and right ROI were also calculated (Table S11).

In supplementary analyses, correlations were assessed between left and right ROI PC_scan2_ and PC_Final_ and clinical variables of interest (Table S12). Outcomes of interest were baseline attenuated positive and negative symptoms measured on the Scale of Prodromal Symptoms (SOPS), verbal memory on the Hopkins Verbal Learning Test- Revised (HVLT-R) [38], and symbol coding on the Brief Assessment of Cognition in Schizophrenia (BACS) [39], as well as the difference in scores between baseline and final assessment for each measure. Protective variables that may partially account for attenuated cortical thinning among CHR-NC relative to CHR-C were also assessed (Table S13). Protective factors included indicators derived from the clinician-administered Structured Assessment of Violence Risk in Youth [40] (SAVRY; prosocial involvement, strong social support, strong attachment and bonds, positive attitude toward intervention/authority, strong commitment to school, resilient personality traits), as well as maternal and paternal education. Multiple linear regression models were conducted among CHR-NC to predict PC_scan2_ and PC_Final_ in the left and right ROIs by each indicator (separately), including age at first scan, age^2^, sex, and scanner as covariates.

## Results

Table 1 presents participant demographic and symptom characteristics by group. Clinical groups did not significantly differ on sex, race/ethnicity, age, or income. HC participants completed one additional scan, on average, compared to both CHR groups. CHR-NC participants were less impaired on baseline clinical measures and a smaller proportion were prescribed antipsychotic medications compared with CHR-C participants.

### Clinical outcome is associated with cortical thinning rate

**Fig. 1:**
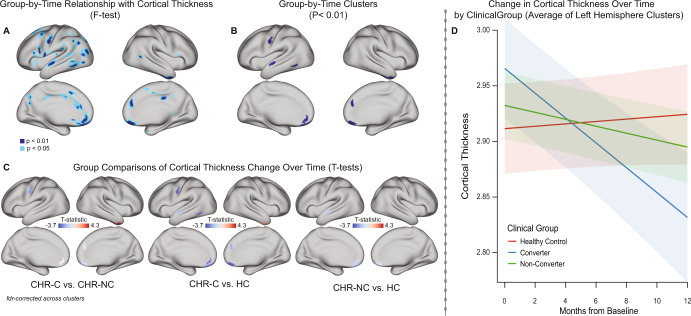
Linear mixed effect models of group-by-time relationships with rate of cortical thickness change. LME models described in parts A-D include age, age^2^, sex, and scanner as fixed effect covariates and a random subject-specific intercept. **A** F-test maps of group-by-time relationships with cortical thickness thresholded at p < 0.01 and p < 0.05 (uncorrected) indicate bilateral areas (described in the text) in which the rate of cortical thickness differs by clinical group. **B** 7 ROIs were retained for further analyses, by clustering areas from part A that pass the p < 0.01 threshold and are at least 100 mm^2^ in area. **C** T-tests (two-sided) across clusters (FDR corrected) to further assess relationships between cortical thickness change in each pair of groups indicate that cortical thinning occurs at a steeper rate in the CHR-C group, compared to the CHR-NC and HC groups in the clusters shown in blue in the image above. In the CHR-NC group, cortical thinning occurs at a steeper rate compared to the HC group in three clusters. **D** To further visualize rates of cortical thickness change by group, the average cortical thickness of left hemisphere clusters (left ROI) is plotted by time from baseline separately for each group. Plot indicates that on average, CHR-C participants have steeper cortical thinning compared to CHR-NC and HC participants.

In initial vertex-level LME models of longitudinal cortical thickness change (including age, age^2^, sex, and scanner as covariates), applying a p < 0.01 (uncorrected) threshold to the F-test map indicated that the clinical group-by-time interaction was significant in bilateral medial orbitofrontal and superior frontal cortex, right posterior cingulate and middle temporal cortex, and left caudal anterior cingulate and additional aspects of the left lateral frontal, temporal, and parietal cortex (Fig. 1A). Applying a more liberal p < 0.05 threshold (uncorrected) indicated that regions identified at p < 0.01 are likely part of larger continuous areas showing the same pattern of association with outcome.

Clusters were derived from F-test maps thresholded at p < 0.01 with at least 100 mm^2^ area (Fig. 1B). Permutation analyses indicated that the p-values of all identified clusters were significant (p ≤ 0.004 for all left hemisphere clusters and p ≤ 0.037 for all right hemisphere clusters). The group-by-time effect on cortical thinning was then assessed in cluster-level LME models (Fig. 1C). Table S7 summarizes group-by-time effects in LME analyses. CHR-C participants had a steeper rate of cortical thinning compared to CHR-NC (in 5 of 7 clusters) and HC participants (in 6 of 7 clusters), and a lower rate of cortical thinning compared to these groups in the right temporal pole cluster. Additionally, CHR-NC participants had a steeper rate of cortical thinning compared with HC participants (in 3 of 7 clusters). FDR-corrected group differences persisted in cluster-based analyses at the p < 0.05 threshold (Figure S2). The directionality of group-by-time effects was the same across left hemisphere clusters comprising the left ROI. In the left ROI, CHR-C participants had the steepest rate of cortical thinning, followed by CHR-NC. In the HC group, cortical thickness in these areas increased slightly (though non-significantly; main effect of time in the HC group: T = 1.29, p = 0.20) across the period of study (Fig. 1D). Left ROI cortical thickness did not differ significantly by group at baseline (F = 1.98, p = 0.14).

When assessing antipsychotic medication as an additional predictor, higher dosage (CPZ equivalents) [41] was significantly associated with lower cortical thickness across time in two left hemisphere clusters, as well as in the left ROI. However, including medication as an additional covariate did not alter the strength or significance of group-by-time effects described in Fig. 1C in 25 of 27 group-by-time comparisons (Table S7).

### Accelerated cortical thinning across less than 3 months is associated with conversion to psychosis

Percent change in thickness (PC) was assessed in the left and right ROIs among the 57 HC, 246 CHR-NC, and 37 CHR-C participants who completed at least two scans. Models predicting PC by clinical group, age, age^2^, sex, and scanner were run separately across first and second scans (PC_scan2_; 2.93±1.81month interscan interval) and first and last scans (PC_Final_; 6.80±2.50 month interscan interval).

Across both time points in the left ROI, thickness decreased at a significantly steeper rate for CHR-C compared to CHR-NC (PC_scan2_: T = −2.12, p_fdr_ = 0.03; PC_Final_: T = −4.28, p_fdr_ < 0.001), CHR-C compared to HC (PC_scan2_: T = −3.55, p_fdr_ < 0.001; PC_Final_: T = −5.27, p_fdr_ < 0.001), and CHR-NC compared to HC (PC_scan2_: T = −2.54, p = 0.02; PC_Final_: T = −2.44, p = 0.03).

In the right ROI, no group differences emerged in PC_scan2_ analyses (Table S8). Between first and last scan, thickness decreased at a significantly steeper rate for CHR-C compared to CHR-NC (PC_Final_; T = −2.17, p_fdr_ = 0.03) and CHR-C compared to HC (T = −2.94, p_fdr_ = 0.003), but not for CHR-NC compared to HC (T =−1.63, p_fdr_ = 0.10).

**Fig. 2:**
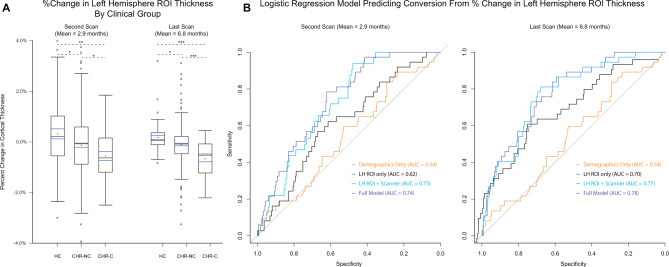
Percent change in left ROI cortical thickness predicts conversion to psychosis. **A** Percent change in cortical thickness in the left ROI between first and second scan (left) and first and last scan (right) indicate a stepwise pattern in which CHR-C < CHR-NC < HC (i.e., most negative percent change in the CHR-C group). Median (black line), mean (blue dot) and SE (blue lines) estimates are added to each box plot. **B** ROC curves predicting conversion among CHR participants from left ROI percent change in cortical thickness, scanner, and demographic covariates (i.e. age, age^2^, and sex) has good classification accuracy (purple line) at second scan (left) and at last scan (right). Comparing this ROC to models with only demographic predictors (orange), only left ROI percent change (black) and left ROI percent change and scanner (blue) indicates that the predictive power of the model is driven primarily by percent change in the left ROI region, not demographic predictors.

PC_scan2_ and PC_Final_ values in the left ROI for each clinical group are presented in Fig. 2A. Including antipsychotic medication dosage as a covariate did not alter the strength or significance of clinical group effects in either ROI, and there was no significant effect of medication on PC_scan2_ or PC_Final_ (see Table S8; Fig. S5).

Among CHR participants (N = 283) a logistic regression model predicting conversion based on left ROI PC_scan2_, age, age^2^, sex, and scanner had an AUC of 0.74 (95% CI from 10,000 bootstrap replications: [0.72, 0.85]). The PC_Final_ model had an AUC of 0.78 (95% CI: [0.75, 0.88]). A model predicting conversion from only left ROI PC_scan2_ and scanner had an AUC of 0.73 (PC_Final_ AUC = 0.77), the AUC for a model with left ROI PC_scan2_ alone was 0.62 (PC_Final_ AUC = 0.73), and a model predicting conversion from demographic variables alone (age, age^2^, and sex) was 0.54 (Fig. 2B). Taken together, models indicate that left ROI PC, not demographic variables, are most predictive of conversion, with PC_Final_ slightly outperforming PC_scan2_ in predicting conversion. See Table S14 for all model coefficients. Mean time to conversion among CHR-C participants included in the percent change analyses was 8.8±7.6 months from first scan. PC_scan2_ and PC_Final_ analyses included 33 pre-conversion and 4 post-conversion CHR-C scans. Repeating LME and percent change analyses excluding post-conversion scans did not alter the strength or significance of the reported results (Table S15).

## Discussion

This study provides the first evidence to-date of steeper cortical thinning among CHR-C prior to psychosis onset, a pattern that was evident across a brief follow-up interval (<3 months) and was predictive of psychosis conversion at the individual subject level.

Results of LME models indicated accelerated cortical thinning among CHR-C in cortical areas previously identified as thinning at a faster rate pre- to post-conversion in individuals who develop psychosis [1–6, 42, 43], including medial orbitofrontal, superior frontal, anterior cingulate, middle and inferior temporal, and parietal cortices. That future converters show a steeper rate of cortical thinning in these regions prior to onset argues for a role of grey matter reduction in the emergence of psychosis, rather than as a consequence of it. Also consistent with this interpretation is the fact that antipsychotic medication exposure did not account for group differences in cortical thinning in this study. However, future work is needed to establish if grey matter changes are a consequence of disease-related neurobiological processes that occur earlier in life.

The present findings point to the potential utility of considering cortical thinning as a biomarker of psychosis-related outcomes. Several models using clinical and demographic measures have predicted conversion among CHR individuals [44–46], at a performance level similar to that based on rate of cortical thinning in this report. Symptom severity and cognitive functioning (baseline and change over time) were not significantly associated with PC_scan2_ or PC_Final_ (Table S12), suggesting that PC scores account for unique predictive power in conversion risk above and beyond symptom impairment. Existing psychosis risk calculators may be improved by incorporating PC in cortical areas identified in this report along with clinical and demographic predictors [47].

In contrast to NAPLS2 findings [1], left hemisphere effects were more prominent in this cohort, with conversion-related cortical thinning present in the left ROI, but not right ROI, for PC_scan2_ analyses and smaller effect sizes compared to prior work in NAPLS2 in right hemisphere regions in which PC was previously linked with conversion [1]. However, in PC_Final_ analyses, thinning across the right ROI was significantly greater among CHR-C compared to CHR-NC and HC. PC_Final_ effect sizes in the left ROI (average interscan interval 6.8 months) were comparable but larger than effect sizes in right hemisphere regions in NAPLS2 with PC calculated across approximately one year. Together with significant PC_scan2_ findings, this may suggest that high-frequency neuroimaging closely following ascertainment is beneficial in identifying individuals at highest risk for conversion.

Although prior work has observed thinner cortex in CHR-NC compared with HC cross-sectionally [42, 43, 48], to our knowledge this is the first study indicating accelerated cortical thinning over time also in CHR-NC compared to HC, albeit at a rate significantly slower than that in CHR-C cases. The difference in the CHR-NC group’s PC scores relative to HC did not change between PC_scan2_ and PC_Final_ calculations, whereas PC slopes of CHR-C relative to CHR-NC and HC became considerably more pronounced during this period. This pattern suggests that CHR-NC experience an initial period of increased cortical thinning close to the point of ascertainment which may subsequently stabilize, potentially helping to explain the high prevalence of persistent subclinical psychotic experiences or other psychiatric impairments among many CHR-NC [49–52].

Protective factors of interest were not associated with PC scores among CHR-NC. Notably, CHR-C and CHR-NC had similar scores on these measures (Table S13). Given known associations between environmental risk factors (e.g., poverty, trauma, low social support) and psychosis-related outcomes [53–55], future in-depth investigations are needed to identify risk and protective factors that may exacerbate or mitigate illness-related neurodevelopmental and symptom outcomes.

Pre-conversion differences in cortical thinning rates among CHR-C may suggest that mechanisms underlying cortical thinning merit investigation as targets of novel pharmaceutical treatments. Prior work implicates atypical synaptic plasticity [7, 15–18], excessive neuroinflammation [19–22], oxidative stress [56, 57], and NMDAR dysfunction [15, 58, 59] as mechanisms contributing to cortical thinning. Higher levels of proinflammatory cytokines among NAPLS2 participants were associated with steeper rates of prefrontal cortical thinning, and to a significantly greater extent among CHR-C, compared to CHR-NC and HC [1]. Additionally, a recent PET study provides direct in-vivo evidence that synaptic density is lower among individuals with schizophrenia, compared to healthy comparison individuals [60].

An important question remains as to the extent to which illness-related cortical thinning can be attenuated or reversed. For example, positive treatment outcomes among individuals with recent onset psychosis who received targeted cognitive training have been associated with attenuated cortical thinning, compared to individuals who did not receive intervention [61]. Future work may benefit from investigating if attenuated cortical thinning among CHR could be a potential mechanism of action for psychotherapeutic interventions.

There are several limitations to the present findings. CHR participants enroled in the study at different points in their prodromal symptom history and thus it was not possible to investigate temporal patterns of cortical thinning starting at symptom onset. The ideal interval in which cortical thickness changes may best serve as a biomarker of psychosis onset may also vary across the age span included in this study. Future work will also benefit from external validation of cortical thinning patterns identified in this report, especially in individualized outcome prediction among CHR cases.

## Conclusions

We identified several cortical areas in which accelerated grey matter reduction across a brief period serves as a risk indicator prior to psychosis onset. Results indicate the importance of evaluating cortical thinning as a biomarker of conversion and encourages further research into mechanisms underlying cortical thinning among CHR individuals to facilitate the development of novel, targeted drug treatments.

## Supplementary information

Supplemental Materials

## Data Availability

Results in this report are derived from publicly available statistical packages in R (v.3.5.3), MATLAB (2017b) and HCP Workbench Command (v.1.5.0).
