# Supplementary material for: Accelerated cortical thinning precedes and predicts conversion to psychosis: The NAPLS3 longitudinal study of youth at clinical high-risk
Source: Mol Psychiatry. 2022 Nov 25;28(3):1182–9. doi: 10.1038/s41380-022-01870-7 (PMC10005940; doi:10.1038/s41380-022-01870-7)
Supplement: Supplementary file 1 — Supplemental Materials [file 41380_2022_1870_MOESM1_ESM.docx]

**Supplemental Materials**

**MRI Acquisition Parameters** (*Page 2*)

Table S1

**MRI Quality Control Procedures** (*Page 2-4*)

Table S2

**Demographics of Excluded Participants who Completed MRI** *(Page 4-5)*

Table S3, S4

**MRI Reliability Study** *(Page 6-9)*

Table S5, S6, Figure S1

**Permutation Testing and LME Results at p<0.05 vs. p<0.01** *(Page 10-11)*

Figure S2

**Thickness across Time** *(Page 11)*

Figure S3

**Medication Analyses** *(Page 12-17)*

Figure S4, S5, Table S7, S8, S9

**Subcortical Analyses** *(Page 18-20)*

Table S10

**Effect Sizes and Comparisons with NAPLS2** *(Page 20-21)*

Table S11

**Symptom Outcomes and Protective Measures** *(Page 21-23)*

Table S12, S13

**Receiver Operating Characteristic Curves** *(Page 24-25)*

Table S14

**Pre-Conversion Only Results** *(Page 26)*

Table S15

MRI Acquisition Parameters

All scans were collected in the sagittal plane with 1mm slice thickness, 256mm x 256 mm matrix, and a 256 mm field of view using a magnetization prepared rapid acquisition gradient-echo (MPRAGE) sequence. Other parameters varied slightly between Siemens and GE manufacturers, as reported in **Table S1**.

| **Siemens (Sites 1, 2, 3, 4 (partial), 5, 8, 9)** | **GE (Sites 4 (partial), 6, 7)** |
| --- | --- |
| Repetition time (TR): 2400 ms  Echo time (TE): 1.96 ms  Inversion time (TI): 1000 ms  Flip angle: 8 degrees | Signa HDxt  Repetition time (TR): 4.27 ms  Echo time (TE): 1.76 ms  Inversion time (TI): 650 ms  Flip angle: 11 degrees  Discovery MR750  Repetition time (TR): 4.00 ms  Echo time (TE): 1.33 ms  Inversion time (TI): 650 ms  Flip angle: 11 degrees  Discovery MR750  Repetition time (TR): 4.64 ms  Echo time (TE): 2.00 ms  Inversion time (TI): 650 ms  Flip angle: 11 degrees |

**Table S1**. Several acquisition parameters varied by scanner manufacturer, as described in the table. Note: Values for Siemens scanners apply to Prisma, Prisma Fit, and Skyra models. Siemens Trio Tim scanners have identical parameters except TE=1.90 ms.

MRI Quality Control Procedures

All T1 MR images were inspected visually by at least two highly trained raters. In cases of discrepant ratings, an additional rater examined the image and provided a third and final rating. Each image was rated on a 0-3 scale as follows:

0= a severe T1 acquisition issue that cannot be improved through manual editing in FreeSurfer, thereby rendering the data unusable.

1= a severe problem with segmentation and/or surface projection rendering the data unusable in its current form; data may be improved by re-running FreeSurfer and manual editing.

2= at least one noticeable problem with segmentation and/or surface projection; data is usable in its current form as a template for BOLD registration but not for T1 structural analyses.

3= high quality data with no noticeable problems with the segmentation and/or surface projection; data is usable in its current form for T1 structural analyses.

A total of 2062 images were rated using this scale. In final consensus ratings, 7 images (0.3%) were rated a 0, 85 (4.1%) were rated a 1, 272 (13.2%) were rated a 2, and 1698 (82.3%) were rated a 3. Only images with a rating of 3 were considered for inclusion in this report.

To further ensure data quality beyond visual inspection, an additional quantitative quality control procedure was implemented to remove scans with extreme outlier cortical thickness values. Scans in which more than 1% of cortical vertices were greater than ± 4 SD from the mean thickness for that vertex across participants were excluded from subsequent analyses. This step removed a total of 10 scans from analyses. One additional session was removed from analyses because no other scans were collected on the same scanner. **Table S2** provides information on the number and percentage of scans passing visual and quantitative QC by scanner.

| **Site** | **Scanner** | **Channels** | **Number of Total Scans** | **Number of Scans Passing Visual and Quantitative QC (% Total)** |
| --- | --- | --- | --- | --- |
| 1 | Siemens Prisma Fit* | 32 | 163 | 153 (93.9%) |
| 1 | Siemens Trio Tim | 32 | 11 | 11 (100%) |
| 2 | Siemens Trio Tim* | 32 | 188 | 170 (90.4%) |
| 3 | Siemens Trio Tim* | 32 | 122 | 109 (89.3%) |
| 4 | GE Signa HDxt | 8 | 53 | 17 (32.1%) |
| 4 | Siemens Prisma* | 64 | 124 | 115 (92.7%) |
| 5 | Siemens Biograph mMR | 12 | 33 | 32 (97.0%) |
| 5 | Siemens Prisma Fit* | 32 | 189 | 187 (98.9%) |
| 5 | Siemens Trio Tim | 32 | 65 | 63 (96.9%) |
| 6 | GE Discovery MR750* | 8 | 272 | 234 (86.0%) |
| 7 | GE Discovery MR750* | 12 | 285 | 101 (35.4%) |
| 8 | Prisma Fit* | 64 | 12 | 12 (100%) |
| 8 | Trio Tim | 32 | 225 | 202 (90.0%) |
| 9 | Siemens Skyra* | 32 | 305 | 277 (90.8%) |

**Table S2.** Data was collected across a total of 14 scanners. Data quality, as indicated by visual and quantitative quality control, indicated that data quality was variable but high overall, with over 90% of scans passing quality control on 11 of the 14 scanners. * indicates scanner that was utilized for the traveling subjects reliability study.

Demographics of Excluded Participants who Completed MRI

Demographics and clinical symptoms were highly similar among participants who completed at least one MRI but were excluded due to quality control considerations, compared with participants included in the main text of this report (Table 1).

| **Characteristic** | **HC**  N=24 | **CHR-NC**  N=150 | **CHR-C**  N=17 | **Statistic** |
| --- | --- | --- | --- | --- |
| Sex (at birth) |  |  |  |  |
| Male | 13 (54%) | 87 (58%) | 8 (47%) | χ^2^ = 0.8, ns |
| Female | 11 (46%) | 63 (42%) | 9 (53%) |  |
| Race/Ethnicity^1^ |  |  |  |  |
| Non-Hispanic white | 11 (46%) | 86 (57%) | 9 (53%) | χ^2^ = 1.2, ns |
| Racial/Ethnic minority | 13 (54%) | 64 (43%) | 8 (47%) |  |
| *N* Taking Antipsychotic Meds^2^ | N/A | 39 (26%) | 11 (65%) | χ^2^ = 9.1, ** |
| *N* No Antipsychotic Meds | N/A | 111 (74%) | 6 (35%) |  |
| *Mean (SD)* |  |  |  |  |
| Age (first scan) | 19.4 (4.2) | 18.2 (3.7) | 19.0 (2.6) | F = 1.2, ns |
| Number of Scans | 4.3 (0.9) | 3.3 (1.7) | 3.4 (1.7) | F = 4.1, * |
| Income^3^ | 4.1 (1.7) | 4.1 (1.8) | 4.1 (1.8) | F = 0.02, ns |
| Total SOPS^4^ Positive Symptoms (Baseline) | 0.9 (1.1) | 12.6 (3.2) | 14.2 (3.0) | F = 162.3, *** |
| Total SOPS Negative Symptoms (Baseline) | 1.0 (1.8) | 11.1 (6.4) | 16.6 (6.2) | F=39.1, *** |
| Global Assessment of Functioning (Baseline) | 84.0 (7.1) | 53.4 (12.4) | 44.2 (4.8) | F=85.4, *** |

**Table S3. Participant characteristics by clinical group among excluded participants.** Healthy control (HC), clinical high-risk non-converter (CHR-NC) and converter (CHR-C) participants who completed at least one MRI but did not meet inclusion criteria for this report were compared on baseline demographic and clinical indicators. P-value terms: ns > 0.05; * < 0.05; ** < 0.01; *** < 0.001. Excluded participants were highly similar to those included in this report within and across groups.

1. Participants self-identified their racial background from one of ten categories: First Nations, East Asian, Southeast Asian, South Asian, Black, Central/South American, West/Central Asia and Middle East, White, Native Hawaiian or Pacific Islander, Interracial. Participants self-identified as non-Hispanic or Hispanic. In this report, racial/ethnic majority refers to non-Hispanic white individuals, whereas racial/ethnic minority refers to Hispanic and/or non-white individuals.
2. Table reflects the number of participants taking antipsychotic medication at the time of at least one neuroimaging visit.
3. Participants self-identified their household income before taxes on a 1-7 scale: 1 = less than $10,000, 2 = $10,000 to $19,999, 3 = $20,000 to $39,999, 4 = $40,000 to $59,999, 5 = $60,000 to $99,999, 6 = $100,000 and above, and 7 = Don’t know or refused to answer. Participants who did not report their income were excluded from mean/SD calculations.
4. The Scale of Prodromal Symptoms (SOPS) is a 19-item scale embedded within the SIPS [1] that assesses four domains of attenuated psychotic symptoms—Positive, Negative, Disorganization, and General Symptoms.

| **Characteristic** | **HC**  N=24 | **CHR-NC**  N=150 | **CHR-C**  N=17 |
| --- | --- | --- | --- |
| Sex (at birth) |  |  |  |
| Male | χ^2^ = 0.01, ns | χ^2^ = 0.2, ns | χ^2^ = 0.3, ns |
| Female |  |  |  |
| Race/Ethnicity |  |  |  |
| Non-Hispanic white | χ^2^ = 0.01, ns | χ^2^ = 3.1, ns | χ^2^ = 0.00, ns |
| Racial/Ethnic minority |  |  |  |
| *N* Taking Antipsychotic Meds | N/A | χ^2^ = 0.1, ns | χ^2^ = 0.3, ns |
| *N* No Antipsychotic Meds | N/A |  |  |
| *Mean (SD)* |  |  |  |
| Age (first scan) | T= -0.1, ns | T= 2.7, ** | T= 0.7, ns |
| Number of Scans | T= -2.5, * | T= -3.7, *** | T= -1.6, * |
| Income | T= -0.2, ns | T= 0.5, ns | T= 0.3, ns |
| Total SOPS Positive Symptoms (Baseline) | T= 0.8, ns | T= 0.7, ns | T= 0.1, ns |
| Total SOPS Negative Symptoms (Baseline) | T= 0.9, ns | T= 1.6, ns | T= -0.9, ns |
| Global Assessment of Functioning (Baseline) | T= 2.4, * | T= -2.1, * | T= 0.3, ns |

**Table S4.** Participant characteristics of included vs. excluded participants. Participants included in this report were compared by group—healthy control (HC), clinical high-risk non-converter (CHR-NC) and converter (CHR-C)—to participants who completed at least one MRI but did not meet inclusion criteria for this report. In all groups, excluded participants completed more scans than included participants. CHR-NC excluded participants were younger and had higher baseline Global Assessment of Functioning (GAF) scores compared with included CHR-NC participants. Excluded HC participants had lower GAF scores relative to included HC participants. There were no other significant demographic or clinical differences between excluded and included participants. All clinical and demographic characteristics are defined in Table S3. P-value terms: ns > 0.05; * < 0.05; ** < 0.01; *** < 0.001.

MRI Reliability Study

Cortical and subcortical regions vary in terms of the reliability of thickness and volume estimates, respectively [2]. Furthermore, in multisite MRI studies it is important to assess variability caused by different scanner hardware, software, and acquisition parameters [3]. To assess test-retest and between-site reliability, NAPLS-3 conducted a traveling subjects study in which each of the 9 sites recruited one healthy subject (5 male, 4 female) ages 19-29 (mean=23.2, SD=3.4), who were scanned on two consecutive days at every site for a total of 162 scans (9 subjects x 9 sites x 2 scans). Scans were completed between May and August 2018 and equipment and software remained the same at each site during this time. Each subject completed all of their scans within two months and the order of site visits were counterbalanced across participants.

Intraclass correlations (ICC) are commonly used as a measure of reliability. ICC is calculated as variance of interest/total variance, where each variance component is quantified by mean squares, which is the sum of squares divided by the number of degrees of freedom [4]. Calculations were performed in R using the VCA package. Subject, site, and session were fully crossed and therefore the total variance of the dependent variable (cortical thickness or subcortical and ventricular volume) was divided into variance attributable to subject, site, session, subject-by-site, subject-by-session, site-by-session, and subject-by-site-by-session. The primary objective of the traveling subjects study was to ascertain sensitivity to detect differential change in cortical thickness and subcortical and ventricular volume over time, where site and session-related variance is not of interest. Therefore, the ICC was calculated as follows, where “V” refers to variance denoted by the subscript:

ICC = (V_Subject_) / Total Variance

The results of this ICC calculation indicate the reliability of cortical thickness and subcortical and ventricular volume measures after considering nuisance variance due to scanner and session. The mean ICC across all regions and scanners was 0.72. To determine how each scanner affected ICC, a leave-site-out calculation was performed by obtaining the ICC for each combination of 8 scanners with 1 scanner removed. Removing the scanner used at sites 6 and 7 was each found to increase the ICC considerably. When removing data from both of these scanners, the mean ICC across all regions in the remaining scanners was 0.88 (a 22% increase). **Table S5** and **Figure S1** show the ICC for all available FreeSurfer-derived estimates of cortical thickness and subcortical and ventricular volumes averaged across all 9 scanners and averaged across 7 scanners after removing the scanners that significantly decreased reliability estimates. Across most regions, ICC coefficients were significantly higher in calculations with 7 scanners compared with the full set of 9 scanners. Given that the key aim of this study is the accurate and reliable calculation of true changes in cortical thickness and subcortical and ventricular volume over time, data from the scanners at sites 6 and 7 were excluded from analyses. Additionally, one scanner at site 4 was discontinued from use approximately one year into data collection, before the traveling subjects study. No reliability data was available for this scanner and only 17 scans (32% of the total) passed visual QC. Therefore, data from this scanner were also excluded from analyses. Overall, a total of 352 scans that passed visual and quantitative QC were removed from analyses. Notably, the three scanners removed due to reliability concerns were also those with the lowest proportion of scans that passed visual QC (see **Table S2**). Intraclass correlations were also calculated separately by scanner, in order to obtain within-scanner estimates of test-retest reliability (**Table S6**).

| **Region** | **All Scanners** | | **Two Scanners Removed** | |
| --- | --- | --- | --- | --- |
|  | **Left** | **Right** | **Left** | **Right** |
| Banks of the Superior Temporal Sulcus | 0.873 | 0.937 | 0.922 | 0.969 |
| Caudal Anterior Cingulate | 0.855 | 0.961 | 0.866 | 0.970 |
| Caudal Middle Frontal | 0.683 | 0.608 | 0.746 | 0.810 |
| Cuneus | 0.812 | 0.272 | 0.936 | 0.773 |
| Entorhinal Cortex | 0.523 | 0.670 | 0.745 | 0.920 |
| Fusiform | 0.727 | 0.644 | 0.928 | 0.930 |
| Inferior Parietal | 0.903 | 0.915 | 0.924 | 0.959 |
| Inferior Temporal | 0.957 | 0.932 | 0.965 | 0.970 |
| Isthmus of Cingulate | 0.941 | 0.949 | 0.965 | 0.981 |
| Lateral Occipital | 0.369 | 0.557 | 0.697 | 0.897 |
| Lateral Orbitofrontal | 0.726 | 0.758 | 0.690 | 0.795 |
| Lingual | 0.497 | 0.881 | 0.795 | 0.967 |
| Medial Orbitofrontal | 0.734 | 0.861 | 0.712 | 0.907 |
| Middle Temporal | 0.822 | 0.622 | 0.965 | 0.929 |
| Parahippocampal | 0.965 | 0.956 | 0.959 | 0.961 |
| paracentral | 0.783 | 0.574 | 0.870 | 0.867 |
| Pars Opercularis | 0.946 | 0.909 | 0.943 | 0.937 |
| Pars Orbitalis | 0.784 | 0.617 | 0.898 | 0.906 |
| Pars Triangularis | 0.917 | 0.908 | 0.907 | 0.926 |
| Pericalcarine | 0.802 | 0.854 | 0.778 | 0.835 |
| Postcentral Gyrus | 0.683 | 0.647 | 0.907 | 0.922 |
| Posterior Cingulate | 0.884 | 0.693 | 0.919 | 0.917 |
| Precentral Gyrus | 0.556 | 0.472 | 0.809 | 0.789 |
| Precuneus | 0.838 | 0.804 | 0.885 | 0.893 |
| Rostral Anterior Cingulate | 0.485 | 0.702 | 0.653 | 0.840 |
| Rostral Middle Frontal | 0.771 | 0.739 | 0.736 | 0.826 |
| Superior Frontal Gyrus | 0.676 | 0.518 | 0.667 | 0.610 |
| Superior Parietal Gyrus | 0.825 | 0.876 | 0.904 | 0.938 |
| Superior Temporal Gyrus | 0.557 | 0.474 | 0.857 | 0.854 |
| Supramarginal Gyrus | 0.936 | 0.966 | 0.971 | 0.969 |
| Frontal Pole | 0.555 | 0.507 | 0.824 | 0.906 |
| Temporal Pole | 0.296 | 0.115 | 0.940 | 0.835 |
| Transverse Temporal Cortex | 0.814 | 0.525 | 0.952 | 0.894 |
| Insula | 0.604 | 0.609 | 0.769 | 0.724 |
| Hippocampus | 0.958 | 0.960 | 0.976 | 0.981 |
| Amygdala | 0.749 | 0.664 | 0.927 | 0.838 |
| Accumbens | 0.232 | 0.503 | 0.944 | 0.876 |
| Lateral Ventricle | 0.996 | 0.997 | 0.998 | 0.998 |
| Thalamus | 0.647 | 0.842 | 0.901 | 0.966 |
| Caudate | 0.962 | 0.965 | 0.988 | 0.978 |
| Putamen | 0.325 | 0.393 | 0.936 | 0.940 |
| Pallidum | 0.447 | 0.422 | 0.886 | 0.918 |
| 3^rd^ Ventricle | 0.928  0.983  0.902 | | 0.990  0.989  0.983 | |
| 4^th^ Ventricle |  |  |  |  |
| Brain Stem |  |  |  |  |

**Table S5**. Values indicate intraclass correlations (ICCs) for FreeSurfer-derived cortical thickness and subcortical and ventricular volume measures of interest, separated by hemisphere and calculated for all 9 scanners and after removing 2 scanners. Intraclass correlations improve considerably when excluding 2 scanners with comparatively low reliability.

**Figure S1.** Boxplots compare intraclass correlations (ICCs) across Freesurfer-derived cortical and subcortical regions (each region represented by a point on the plot) in all 9 scanners tested in the traveling participants study (left) vs. after excluding two scanners with poorer reliability (right). The mean ICC increased from 0.72 in all scanners to 0.88 after removing two scanners. Plots include mean ICC (blue dot), standard error (blue lines), and median (black line).

| **Site 1** | **Site 2** | **Site 3** | **Site 4** | **Site 5** | **Site 6** | **Site 7** | **Site 8** | **Site 9** |
| --- | --- | --- | --- | --- | --- | --- | --- | --- |
| 0.934 | 0.935 | 0.913 | 0.924 | 0.944 | 0.856 | 0.794 | 0.935 | 0.906 |

**Table S6**. Values indicate the average intraclass correlation (ICC) for FreeSurfer-derived cortical thickness and subcortical and ventricular volume measures, calculated separately by scanner. These test-retest ICCs were excellent (greater than 0.9) for most scanners, and lower in two scanners (Sites 6 and 7) from which data were excluded for this report. Information on the scanner used in the traveling participants reliability study for each site is detailed in Table S2.

Permutation Testing and LME Results at p<0.05 vs. p<0.01

In primary analyses, clusters were formed from F-test maps of group-by-time relationships with cortical thickness from LME analyses thresholded at p<0.01 (Figure 1). Permutation testing assessed the significance of each cluster at the p<0.01 threshold by repeating LME analyses on 1,000 bootstrap replicates in which data were permutated by group labels only. Permutation testing results indicated p≤0.04 for all left hemisphere clusters (LH cluster 1: p=0.001, LH cluster 2: p=0.002, LH cluster 3: p=0.004, LH cluster 4: p=0.004) and p≤0.037 for all right hemisphere clusters (RH cluster 1: 0.037, RH cluster 2: p=0.005, RH cluster 3: p<0.001).

All group comparisons of cortical thickness change over time were FDR-corrected across clusters. Thresholding F-test maps at p<0.05 yields 26 clusters (Figure 1a). When applying FDR-correction across clusters, CHR-C participants had a steeper rate of cortical thinning compared to HC (in 25 of 26 clusters, as shown in **Figure S2**). CHR-C had accelerated thinning compared to CHR-NC (in 24 of 26 clusters), and CHR-NC did not have accelerated thinning compared to HC in any clusters.

**Figure S2.** Comparing FDR-corrected group differences in rates of cortical thinning over time in 26 clusters derived from F-test maps thresholded at p<0.05 indicates a similar pattern to cluster results derived from p<0.01 maps presented in the main text, indicating that correcting across a larger number of clusters derived from a looser statistical threshold does not substantially alter observed effects. Figure shows CHR-C vs. HC maps (two-sided T-test), the contrast in which the highest number of clusters survive correction. Of note, CHR-NC vs. HC comparisons do not pass FDR-correction in p<0.05 cluster analyses.

Thickness across Time


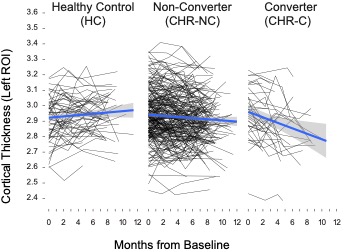


**Figure S3**. Thickness across time in the left ROI is shown for healthy control (HC), clinical high-risk non-converter (CHR-NC) and converter (CHR-C) participants, with the linear trend line shown (in blue) for each group. N=120 participants completed 1 scan, N=78 completed 2 scans, N=100 completed 3 scans, N=80 completed 4 scans, and N=82 completed 5 scans that met quality control standards for inclusion in this report.

Medication Analyses

Antipsychotic medications have previously been linked with gray matter decline in individuals with schizophrenia [5, 6]. To evaluate the effects of antipsychotic medications, all LME and percent change models presented in the main text were compared to models including antipsychotic medication dose as a covariate (in addition to all other covariates listed in the main text). Antipsychotic medication was not included as a covariate in primary analyses given the overlap between medication dosage and clinical outcome (i.e. participants who ultimately convert to psychosis are more likely to be prescribed antipsychotic medications due to illness severity). Specifically, significantly more CHR-C participants (52%) were prescribed antipsychotic medications during the period of study, compared with CHR-NC participants (28%; Table 1). Antipsychotic medication dose *at each scan date* was included as a covariate in LME analyses. For percent change analyses, the average antipsychotic medication dose between first scan and second scan (for PC_scan2_) or last scan (for PC_Final_) was calculated. All antipsychotic medication dosages were calculated in chlorpromazine (CPZ) equivalent units, for comparison across drugs [7].

Clusters are labeled in **Figure S4** for reference. In LME models, group-by-time effects are largely unchanged when antipsychotic medication is added as a covariate. In the combined right ROI for the CHR-C vs. HC group-by-time comparison and left hemisphere cluster 2 for the CHR-C vs. CHR-NC group-by time comparison, adding antipsychotic medication as a covariate changed the group-by-time association from p<0.05 to a non-significant trend. In all other clusters and group comparisons, antipsychotic medications did not alter the significance of group-by-time effects. Medication was associated with lower cortical thickness in left hemisphere clusters 3, 4, and the left ROI **(Table S7)**. In percent change models (PC_scan2_ and PC_Final_), including antipsychotic medication as a covariate did not alter the significance of group effects in the left or right ROI. Medication did not significantly affect percent change in any PC_scan2_ or PC_Final_ calculations (**Table S8**). Group comparisons in LME and percent change models for the left ROI with and without medications included as a covariate are presented visually in **Figure S5.** Finally, average PC_scan2_ and PC_Final_ were compared between CHR-NC participants who were medicated and unmedicated, and for CHR-C medicated and unmedicated participants. PC_scan2_ and PC_Final_ did not differ between CHR-C participants regardless of medication status. PC_scan2_ did not differ between CHR-NC medicated and unmedicated participants, though PC_Final_ was significantly lower in CHR-NC participants who were prescribed antipsychotic medications (N = 71) vs. unmedicated CHR-NC participants (N=175) (**Table S9**). Results across medication analyses suggest that antipsychotic medications do not explain group differences in cortical thickness over time but may affect cortical thinning, in line with prior work [5, 6, 8].

**Figure S4.** Left and right hemisphere clusters used in LME and percent difference analyses are labeled for reference.

| **CHR-NC vs. HC** | | | | | | |
| --- | --- | --- | --- | --- | --- | --- |
| **Region** | **Model Without Medication** | | **Model Including Medication (CPZ Equivalents)** | | | |
|  | T-Statistic  (Group) | P-value  (Group) | T-statistic  (Group) | P-value  (Group) | | Change in Significance of Group Effect in  Model Without Meds vs. Model with Meds |
| RH Cluster 1 | -1.782 | 0.097 | -1.712 | 0.112 | | No |
| RH Cluster 2 | -2.481 | 0.024 | -2.450 | 0.026 | | No |
| RH Cluster 3 | -0.989 | 0.323 | -0.938 | 0.348 | | No |
| Right ROI | -2.606 | 0.021 | -2.555 | 0.024 | | No |
| LH Cluster 1 | -1.287 | 0.223 | -1.196 | 0.261 | | No |
| LH Cluster 2 | -2.784 | 0.016 | -2.765 | 0.022 | | No |
| LH Cluster 3 | -2.053 | 0.061 | -1.939 | 0.079 | | No |
| LH Cluster 4 | -2.810 | 0.016 | -2.682 | 0.022 | | No |
| Left ROI | -3.386 | 0.007 | -3.302 | 0.009 | | No |
| **CHR-C vs. HC** | | | | | | |
| **Region** | **Model Without Medication** | | **Model Including Medication (CPZ Equivalents)** | | | |
|  | T-Statistic  (Group) | P-value  (Group) | T-statistic  (Group) | P-value  (Group) | | Change in Significance of Group Effect in  Model Without Meds vs. Model with Meds |
| RH Cluster 1 | -3.190 | 0.002 | -2.913 | 0.005 | | No |
| RH Cluster 2 | -3.676 | 0.001 | -3.485 | 0.002 | | No |
| RH Cluster 3 | 3.283 | 0.002 | 3.399 | 0.002 | | No |
| Right ROI | -2.090 | 0.037 | -1.860 | 0.063 | | Yes |
| LH Cluster 1 | -3.674 | 0.001 | -3.444 | 0.002 | | No |
| LH Cluster 2 | -3.539 | 0.001 | -3.274 | 0.002 | | No |
| LH Cluster 3 | -3.456 | 0.001 | -3.041 | 0.004 | | No |
| LH Cluster 4 | -3.062 | 0.003 | -2.598 | 0.011 | | No |
| Left ROI | -4.618 | <0.001 | -4.193 | <0.001 | | No |
| **CHR-C vs. CHR-NC** | | | | | | |
| **Region** | **Model Without Medication** | | **Model Including Medication (CPZ Equivalents)** | | | |
|  | T-Statistic  (Group) | P-value  (Group) | T-statistic  (Group) | P-value  (Group) | | Change in Significance of Group Effect in  Model Without Meds vs. Model with Meds |
| RH Cluster 1 | -2.422 | 0.023 | -2.166 | 0.046 | | No |
| RH Cluster 2 | -2.515 | 0.022 | -2.332 | 0.045 | | No |
| RH Cluster 3 | 4.289 | <0.001 | 4.384 | <0.001 | | No |
| Right ROI | -0.666 | 0.505 | -0.453 | 0.651 | | No |
| LH Cluster 1 | -3.277 | 0.005 | -3.086 | 0.009 | | No |
| LH Cluster 2 | -2.164 | 0.039 | -1.890 | 0.076 | | Yes |
| LH Cluster 3 | -2.546 | 0.022 | -2.169 | 0.046 | | No |
| LH Cluster 4 | -1.628 | 0.117 | -1.207 | 0.256 | | No |
| Left ROI | -2.986 | 0.009 | -2.579 | 0.030 | | No |
| **All Tests** | | | | | | |
| **Region** | **Model Including Medication (CPZ Equivalents)** | | | | | |
|  | T-statistic  (CPZ Equivalents) | | | | P-value  (CPZ Equivalents) | |
| RH Cluster 1 | -1.932 | | | | 0.117 | |
| RH Cluster 2 | -1.001 | | | | 0.317 | |
| RH Cluster 3 | -1.012 | | | | 0.317 | |
| Right ROI | -1.466 | | | | 0.184 | |
| LH Cluster 1 | -1.681 | | | | 0.139 | |
| LH Cluster 2 | -1.846 | | | | 0.117 | |
| LH Cluster 3 | -2.678 | | | | 0.023 | |
| LH Cluster 4 | -3.356 | | | | 0.006 | |
| Left ROI | -3.203 | | | | 0.006 | |

**Table S7:** Tables compare group coefficients (Group 1 vs. Group 2 rate of thickness change) in linear mixed effects models that do and do not include antipsychotic medication as a covariate. Results indicate that including medication as a covariate does not alter the significance of group effects (with two exceptions, as noted in the table). There is a significant main effect of medication in left hemisphere clusters 3 and 4, and in the average of all left hemisphere clusters (left ROI), in which medication is linked with lower cortical thickness over time. Medication is not significantly associated with thickness in other clusters. All p-values reflect FDR-corrected estimates across all clusters; all T-tests are two-sided.

| **Percent Difference in Cortical Thickness (CHR-NC vs. HC)** | | | | | | |
| --- | --- | --- | --- | --- | --- | --- |
| **Region** | **Model Without Medication** | | **Model Including Medication (CPZ Equivalents)** | | | |
|  | T-Statistic  (Group) | P-value  (Group) | T-statistic  (Group) | P-value  (Group) | | Change in Significance of Group Effect in  Model Without Meds vs. Model with Meds |
| RH ROI Scan 2 | -1.454 | 0.147 | -1.294 | 0.196 | | No |
| LH ROI Scan 2 | -2.536 | 0.023 | -2.310 | 0.043 | | No |
| RH ROI Final Scan | -1.634 | 0.103 | -1.472 | 0.142 | | No |
| LH ROI Final Scan | -2.438 | 0.031 | -2.317 | 0.042 | | No |
| **Percent Difference in Cortical Thickness (CHR-C vs. HC)** | | | | | | |
| **Region** | **Model Without Medication** | | **Model Including Medication (CPZ Equivalents)** | | | |
|  | T-Statistic  (Group) | P-value  (Group) | T-statistic  (Group) | P-value  (Group) | | Change in Significance of Group Effect in  Model Without Meds vs. Model with Meds |
| RH ROI Scan 2 | -1.105 | 0.270 | -0.932 | 0.352 | | No |
| LH ROI Scan 2 | -3.546 | 0.001 | -3.274 | 0.002 | | No |
| RH ROI Final Scan | -2.944 | 0.003 | -2.669 | 0.008 | | No |
| LH ROI Final Scan | -5.270 | <0.001 | -5.005 | <0.001 | | No |
| **Percent Difference in Cortical Thickness (CHR-C vs. CHR-NC)** | | | | | | |
| **Region** | **Model Without Medication** | | **Model Including Medication (CPZ Equivalents)** | | | |
|  | T-Statistic  (Group) | P-value  (Group) | T-statistic  (Group) | P-value  (Group) | | Change in Significance of Group Effect in  Model Without Meds vs. Model with Meds |
| RH ROI Scan 2 | -0.116 | 0.907 | -0.047 | 0.963 | | No |
| LH ROI Scan 2 | -2.121 | 0.034 | -2.018 | 0.048 | | No |
| RH ROI Final Scan | -2.168 | 0.031 | -2.022 | 0.044 | | No |
| LH ROI Final Scan | -4.284 | <0.001 | -4.160 | <0.001 | | No |
| **Percent Difference in Cortical Thickness (All Tests)** | | | | | | |
| **Region** | **Model Including Medication (CPZ Equivalents)** | | | | | |
|  | T-statistic  (CPZ Equivalents) | | | | P-value  (CPZ Equivalents) | |
| RH ROI Scan 2 | -0.832 | | | | 0.406 | |
| LH ROI Scan 2 | -1.152 | | | | 0.406 | |
| RH ROI Final Scan | -0.868 | | | | 0.593 | |
| LH ROI Final Scan | -0.534 | | | | 0.593 | |

**Table S8:** Tables compare group coefficients (Group 1 vs. Group 2 rate of thickness change) in percent difference in cortical thickness models that do and do not include antipsychotic medication as a covariate. Results indicate that including medication as a covariate does not alter the significance of group effects. There is not a significant main effect of medication in any ROI. All p-values reflect FDR-corrected estimates across all ROIs; all T-tests are two-sided.

**Figure S5.** LME and percent change (PC_scan2_ and PC_Final_) models for the left ROI were compared with and without included antipsychotic medication dosage as a covariate. Models included all other covariates listed in the main text. Antipsychotic medication did not significantly affect the strength of group differences in cortical thickness change in LME or percent change models for the left ROI. In LME models (but not percent change models), antipsychotic medication was associated with thinner cortex over time in the left ROI. Note: all T-statistics are negative (direction flipped on y axis for ease of viewing). All T-tests are two-sided. P-value terms: ns > 0.05; * < 0.05; ** < 0.01; *** < 0.001.

|  | **Mean (Percent Difference)** | **T-Statistic (No Meds vs. Meds)** | **P-value** |
| --- | --- | --- | --- |
| **CHR-NC No Medications**  **Scan 2 (N = 184)** | -0.0002 | 1.744 | 0.084 |
| **CHR-NC Medications Scan 2 (N = 62)** | -0.004 |  |  |
| **CHR-NC No Medications**  **Final Scan (N = 175)** | -0.0003 | 2.490 | 0.014 |
| **CHR-NC Medications Final Scan (N = 71)** | -0.003 |  |  |
| **CHR-C No Medications Scan 2 (N = 21)** | -0.008 | -0.795 | 0.432 |
| **CHR-C Medications**  **Scan 2 (N = 16)** | -0.005 |  |  |
| **CHR-C No Medications Final Scan (N = 18)** | -0.007 | -0.547 | 0.588 |
| **CHR-C Medications**  **Final Scan (N = 19)** | -0.006 |  |  |

**Table S9:** PC_scan2_ and PC_Final_ in the left ROI was compared for participants taking antipsychotic medications and those not taking antipsychotic medications. At both time points, there were no differences in mean percent difference between CHR-C participants who did/did not take medications. There was no significant difference in PC_scan2_ between CHR-NC who did and did not take antipsychotic medications; at final scan CHR-NC participants who took antipsychotic medications had significantly lower PC_Final_ in the left ROI compared to unmedicated participants. Average dosage of antipsychotic medications (in CPZ equivalent units) across first and second scan was 52.5 (SD = 123.0) for CHR-NC participants and 92.9 (SD = 163.9) for CHR-C participants; average dosage across first and last scan was 56.9 (SD = 132.6) for CHR-NC participants and 126.4 (SD = 195.2) for CHR-C participants. All T-tests are two-sided.

Subcortical Analyses

LME models were conducted for subcortical and ventricular volumes of interest, including the bilateral hippocampus, amygdala, accumbens, lateral ventricle, thalamus, caudate, putamen, pallidum, and the 3^rd^ ventricle, 4^th^ ventricle, and brain stem. Longitudinal volume was predicted by the same fixed effects as in cortical thickness models (i.e., time from first scan, age, age^2^, sex, clinical group, clinical group-by-time, scanner), as well as intracranial volume. Models with and without antipsychotic medication dosage included as a covariate were compared. Group-by-time effects for each group-wise comparison and the main effect of medication for each region are presented in **Table S10**, FDR-corrected across regions. Given null findings in LME models, further percent difference analyses were not conducted for subcortical and ventricular volumes.

| **CHR-NC vs. HC** | | | | | |
| --- | --- | --- | --- | --- | --- |
| **Region** | **Model Without Medication** | | **Model Including Medication (CPZ Equivalents)** | | |
|  | T-Statistic  (Group) | P-value  (Group)  FDR-Corrected | T-statistic  (Group) | | P-value  (Group)  FDR Corrected |
| LH Hippocampus | -0.689 | 0.748 | -0.611 | | 0.734 |
| RH Hippocampus | -2.040 | 0.203 | -1.925 | | 0.259 |
| LH Amygdala | -1.106 | 0.568 | -1.099 | | 0.575 |
| RH Amygdala | -0.657 | 0.748 | -0.640 | | 0.734 |
| LH Accumbens | -0.352 | 0.861 | -0.344 | | 0.868 |
| RH Accumbens | -0.446 | 0.831 | -0.472 | | 0.807 |
| LH Lateral Ventricle | 2.307 | 0.202 | 2.213 | | 0.258 |
| RH Lateral Ventricle | 2.030 | 0.203 | 1.953 | | 0.259 |
| LH Thalamus | -0.292 | 0.861 | -0.247 | | 0.871 |
| RH Thalamus | -1.225 | 0.524 | -1.172 | | 0.574 |
| LH Caudate | -0.932 | 0.668 | -0.910 | | 0.690 |
| RH Caudate | -0.593 | 0.751 | -0.638 | | 0.734 |
| LH Putamen | -1.601 | 0.298 | -1.617 | | 0.289 |
| RH Putamen | -0.717 | 0.748 | -0.725 | | 0.734 |
| LH Pallidum | -0.106 | 0.915 | -0.162 | | 0.871 |
| RH Pallidum | -0.161 | 0.915 | -0.177 | | 0.871 |
| 3^rd^ Ventricle | 2.342 | 0.202 | 2.314 | | 0.258 |
| 4^th^ Ventricle | 1.887 | 0.226 | 1.791 | | 0.274 |
| Brain Stem | -1.769 | 0.244 | -1.716 | | 0.274 |
| **CHR-C vs. HC** | | | | | |
| **Region** | **Model Without Medication** | | **Model Including Medication (CPZ Equivalents)** | | |
|  | T-Statistic  (Group) | P-value  (Group)  FDR-Corrected | T-statistic  (Group) | | P-value  (Group)  FDR Corrected |
| LH Hippocampus | -0.566 | 0.678 | -0.249 | | 0.848 |
| RH Hippocampus | -2.099 | 0.229 | -1.723 | | 0.381 |
| LH Amygdala | -0.059 | 0.953 | -0.070 | | 0.944 |
| RH Amygdala | -0.393 | 0.776 | -0.339 | | 0.848 |
| LH Accumbens | -1.857 | 0.242 | -1.698 | | 0.381 |
| RH Accumbens | 0.880 | 0.600 | 0.762 | | 0.652 |
| LH Lateral Ventricle | 2.620 | 0.085 | 2.296 | | 0.208 |
| RH Lateral Ventricle | 1.938 | 0.242 | 1.645 | | 0.381 |
| LH Thalamus | -0.764 | 0.604 | -0.691 | | 0.665 |
| RH Thalamus | -1.530 | 0.401 | -1.408 | | 0.505 |
| LH Caudate | -0.268 | 0.833 | -0.280 | | 0.848 |
| RH Caudate | -2.659 | 0.085 | -2.798 | | 0.100 |
| LH Putamen | 1.110 | 0.508 | 1.044 | | 0.564 |
| RH Putamen | -1.138 | 0.508 | -1.147 | | 0.564 |
| LH Pallidum | -0.790 | 0.604 | -0.964 | | 0.579 |
| RH Pallidum | 0.643 | 0.659 | 0.505 | | 0.778 |
| 3^rd^ Ventricle | 0.957 | 0.585 | 0.837 | | 0.638 |
| 4^th^ Ventricle | 1.419 | 0.424 | 1.094 | | 0.564 |
| Brain Stem | -1.225 | 0.508 | -1.099 | | 0.564 |
| **CHR-C vs. CHR-NC** | | | | | |
| **Region** | **Model Without Medication** | | **Model Including Medication (CPZ Equivalents)** | | |
|  | T-Statistic  (Group) | P-value  (Group)  FDR-Corrected | T-statistic  (Group) | | P-value  (Group)  FDR-Corrected |
| LH Hippocampus | -0.195 | 0.893 | 0.106 | | 0.974 |
| RH Hippocampus | -1.045 | 0.721 | -0.712 | | 0.793 |
| LH Amygdala | 0.635 | 0.769 | 0.611 | | 0.793 |
| RH Amygdala | -0.022 | 0.983 | 0.023 | | 0.982 |
| LH Accumbens | -1.845 | 0.414 | -1.676 | | 0.596 |
| RH Accumbens | 1.262 | 0.721 | 1.145 | | 0.793 |
| LH Lateral Ventricle | 1.456 | 0.692 | 1.171 | | 0.793 |
| RH Lateral Ventricle | 0.874 | 0.721 | 0.610 | | 0.793 |
| LH Thalamus | -0.665 | 0.769 | -0.614 | | 0.793 |
| RH Thalamus | -0.926 | 0.721 | -0.831 | | 0.793 |
| LH Caudate | 0.290 | 0.893 | 0.258 | | 0.974 |
| RH Caudate | -2.582 | 0.190 | -2.714 | | 0.129 |
| LH Putamen | 2.246 | 0.237 | 2.175 | | 0.284 |
| RH Putamen | -0.813 | 0.721 | -0.823 | | 0.793 |
| LH Pallidum | -0.811 | 0.721 | -0.972 | | 0.793 |
| RH Pallidum | 0.817 | 0.721 | 0.673 | | 0.793 |
| 3^rd^ Ventricle | -0.415 | 0.886 | -0.519 | | 0.820 |
| 4^th^ Ventricle | 0.387 | 0.886 | 0.097 | | 0.974 |
| Brain Stem | -0.245 | 0.893 | -0.149 | | 0.974 |
| **All Tests** | | | | | |
| **Region** | **Model Including Medication (CPZ Equivalents)** | | | | |
|  | T-statistic  (CPZ Equivalents) | | | P-value  (CPZ Equivalents)  FDR Corrected | |
| LH Hippocampus | -2.384 | | | 0.165 | |
| RH Hippocampus | -2.601 | | | 0.165 | |
| LH Amygdala | -0.038 | | | 0.970 | |
| RH Amygdala | -0.312 | | | 0.897 | |
| LH Accumbens | -1.465 | | | 0.454 | |
| RH Accumbens | 0.657 | | | 0.702 | |
| LH Lateral Ventricle | 2.080 | | | 0.180 | |
| RH Lateral Ventricle | 1.897 | | | 0.221 | |
| LH Thalamus | -0.648 | | | 0.702 | |
| RH Thalamus | -1.011 | | | 0.659 | |
| LH Caudate | 0.133 | | | 0.946 | |
| RH Caudate | 1.120 | | | 0.625 | |
| LH Putamen | 0.321 | | | 0.897 | |
| RH Putamen | 0.130 | | | 0.946 | |
| LH Pallidum | 1.162 | | | 0.625 | |
| RH Pallidum | 0.868 | | | 0.683 | |
| 3^rd^ Ventricle | 0.850 | | | 0.683 | |
| 4^th^ Ventricle | 2.167 | | | 0.180 | |
| Brain Stem | -0.776 | | | 0.693 | |

**Table S10.** Two-sided t-statistics and FDR-corrected p-values in LME models with and without antipsychotic medication included as a covariate indicate that there are no significant effects of clinical-group by time on longitudinal changes in subcortical and ventricular volumes. There is not a significant main effect of medication on volume in any subcortical or ventricular region.

Effect Sizes and Comparisons with NAPLS2

The effect size of group differences (CHR-C vs. CHR-NC and HC) were larger for PC_Final_ (left: d=-0.83, right: d=-0.41) compared with PC_scan2_ (left: d=-0.49, right: d=-0.11). Effect sizes were larger when comparing CHR-C to HC, with the largest effect (d= -1.22) observed for PC_Final_ in the left ROI. This effect is larger than the maximum effect size (d=-1.0) observed in PC analyses in NAPLS2 [8]. In three regions in which CHR-C were shown to demonstrate accelerated cortical thinning relative to CHR-NC and HC in NAPLS2, effect sizes were smaller than those reported in NAPLS2 [8] (**Table S11**).

| **ROI** | **Cohen’s d**  **CHR-C vs. HC**  **(PC_scan2_)** | **Cohen’s d**  **CHR-C vs. CHR-NC (PC_scan2_)** | **Cohen’s d**  **CHR-NC vs. HC (PC_scan2_)** | **Cohen’s d**  **CHR-C vs. CHR-NC and HC (PC_scan2_)** |
| --- | --- | --- | --- | --- |
| Left ROI | -0.72 | -0.43 | -0.36 | -0.49 |
| Right ROI | -0.27 | -0.07 | -0.24 | -0.11 |
| NAPLS2 Right Medial OFC | -0.35 | -0.13 | -0.26 | -0.18 |
| NAPLS2 Right Middle Frontal | -0.17 | -0.04 | -0.18 | -0.06 |
| NAPLS2 Right Superior Frontal | -0.02 | 0.07 | -0.10 | 0.05 |
|  | | | |  |
| **ROI** | **Cohen’s d**  **CHR-C vs. HC**  **(PC_Final_)** | **Cohen’s d**  **CHR-C vs. CHR-NC (PC_Final_)** | **Cohen’s d**  **CHR-NC vs. HC (PC_Final_)** | **Cohen’s d**  **CHR-C vs. CHR-NC and HC (PC_Final_)** |
| Left ROI | -1.22 | -0.75 | -0.41 | -0.83 |
| Right ROI | -0.72 | -0.35 | -0.31 | -0.41 |
| NAPLS2 Right Medial OFC | -0.41 | -0.23 | -0.20 | -0.27 |
| NAPLS2 Right Middle Frontal | -0.25 | -0.17 | -0.07 | -0.18 |
| NAPLS2 Right Superior Frontal | -0.10 | -0.10 | -0.04 | -0.10 |

**Table S11.** Effect sizes (Cohen’s d) for each group contrast were assessed at PC_scan2_ and PC_Final_ for the left and right ROIs identified in this report. To assess consistency with prior percent change findings in NAPLS2, effect sizes were assessed for PC_scan2_ and PC_Final_ in three regions identified in Cannon et al. 2015. Overall, effect sizes in the NAPLS2 regions (CHR-C vs. CHR-NC and HC) were lower in this report (PC_scan2_ d = 0.05 to -0.18; PC_Final_ d = -0.10 to -0.27) compared to Cannon et al. 2015 (d= -0.30 to -1.00). Effect sizes in the left and right ROI identified in this report were comparable to effect sizes observed in NAPLS2 (PC_scan2_ d = -0.11 to -0.49; PC_Final_ d = -0.41 to -0.83). For all table entries, negative effect sizes indicate that the percent change value is lower in the first group relative to the second group.

Symptom Outcomes and Protective Measures

Clinical symptom and cognitive functioning baseline and change scores were not significantly correlated with PC_scan2_ or PC_Final_ in the right or left ROI (**Table S12**). However, a non-sigifcant trend was present in which higher baseline positive and negative were associated with lower PC_Final_ (positive symptoms: R=-0.16, p_FDR_= 0.06; negative syptoms: R=-0.15, p_FDR_=0.07). Among CHR-NC, no protective factors of interest were associated with PC_scan2_ or PC_Final_ in the right or left ROI (**Table S13**)**.**

| **Outcome** | **Left ROI**  **r (PC_scan2_)** | **Left ROI**  **P-value (PC_scan2_)** | **Right ROI**  **r (PC_scan2_)** | **Right ROI**  **P-value (PC_scan2_)** |
| --- | --- | --- | --- | --- |
| SOPS Positive (BL) | -0.143 | 0.177 | -0.072 | 0.642 |
| SOPS Positive (Difference) | -0.063 | 0.704 | 0.015 | 0.846 |
| SOPS Negative (BL) | -0.109 | 0.279 | -0.055 | 0.716 |
| SOPS Negative (Difference) | -0.034 | 0.733 | -0.025 | 0.765 |
| BACS (BL) | 0.003 | 0.959 | -0.037 | 0.733 |
| BACS (Difference) | -0.048 | 0.716 | 0.122 | 0.258 |
| HVLT (BL) | 0.034 | 0.733 | -0.048 | 0.716 |
| HVLT (Difference) | 0.024 | 0.765 | 0.079 | 0.642 |
|  | | | | |
| **Outcome** | **Left ROI**  **r (PC_Final_)** | **Left ROI**  **P-value (PC_Final_)** | **Right ROI**  **r (PC_Final_)** | **Right ROI**  **P-value (PC_Final_)** |
| SOPS Positive (BL) | -0.161 | 0.064 | -0.116 | 0.202 |
| SOPS Positive (Difference) | -0.068 | 0.524 | -0.001 | 0.993 |
| SOPS Negative (BL) | -0.146 | 0.074 | -0.073 | 0.524 |
| SOPS Negative (Difference) | 0.011 | 0.972 | -0.011 | 0.972 |
| BACS (BL) | -0.041 | 0.690 | -0.041 | 0.690 |
| BACS (Difference) | -0.013 | 0.972 | 0.069 | 0.524 |
| HVLT (BL) | 0.049 | 0.690 | -0.006 | 0.981 |
| HVLT (Difference) | 0.046 | 0.690 | 0.111 | 0.202 |

**Table S12.** Correlation coefficients (Pearson’s R) are calculated between eight outcomes of interest and PC_scan2_ and PC_Final._ After applying FDR correction to account for multiple comparisons across outcomes and hemispheres, no clinical outcome is significantly associated with percent change in cortical thickness at either time point. There is a non-significant trend in which baseline positive and negative symptoms are associated with a steeper percent decrease in cortical thickness at final scan (PC_Final_). Terms: SOPS = Scale of Prodromal Symptoms; BACS = Brief Assessment of Cognition in Schizophrenia; HVLT = Hopkins Verbal Learning Test; BL = baseline; Difference = final score – baseline score.

| **Protective Factor** | **Left ROI**  **T-stat (PC_scan2_)** | **Left ROI**  **P-value (PC_scan2_)** | **Right ROI**  **T-stat (PC_scan2_)** | **Right ROI**  **P-value (PC_scan2_)** | **T-test (CHR-NC vs. CHR-C)** |
| --- | --- | --- | --- | --- | --- |
| Prosocial Involvement | -0.142 | 0.923 | -1.327 | 0.744 | T=1.959, p=0.225 |
| Strong Social Support | 0.643 | 0.784 | 0.261 | 0.923 | T=0.751, p=0.522 |
| Strong Attachment and Bonds | 0.907 | 0.784 | 0.995 | 0.784 | T=1.615, p=0.251 |
| Positive Attitude Toward Intervention/Authority | 1.681 | 0.693 | 0.106 | 0.923 | T=1.442, p=0.251 |
| Strong Commitment to School | -0.097 | 0.923 | -0.616 | 0.784 | T=0.960, p=0.456 |
| Resilient Personality Traits | -0.271 | 0.923 | -1.520 | 0.693 | T=2.112, p=0.225 |
| Education (Father) | 0.649 | 0.784 | 1.582 | 0.693 | T=-1.499, p=0.251 |
| Education (Mother) | 0.772 | 0.784 | 1.028 | 0.784 | T=-0.092, p=0.927 |
|  | | | | | |
| **Protective Factor** | **Left ROI**  **T-stat (PC_Final_)** | **Left ROI**  **P-value (PC_Final_)** | **Right ROI**  **T-stat (PC_Final_)** | **Right ROI**  **P-value (PC_Final_)** |  |
| Prosocial Involvement^1^ | -0.167 | 0.973 | -1.615 | 0.862 |  |
| Strong Social Support | 0.458 | 0.973 | 0.128 | 0.973 |  |
| Strong Attachment and Bonds | -0.284 | 0.973 | -0.634 | 0.973 |  |
| Positive Attitude Toward Intervention/Authority | 2.143 | 0.531 | 0.775 | 0.973 |  |
| Strong Commitment to School | 0.034 | 0.973 | -0.730 | 0.973 |  |
| Resilient Personality Traits | 0.861 | 0.973 | -0.606 | 0.973 |  |
| Education (Father)^2^ | 0.270 | 0.973 | 0.252 | 0.973 |  |
| Education (Mother) | 0.408 | 0.973 | -0.106 | 0.973 |  |

**Table S13.** Exploratory analyses indicate that among CHR-NC, eight protective factors of interest are not significantly associated with PC_scan2_ or with PC_Final_, applying FDR correction to account for multiple comparisons across outcomes and hemispheres. All T-tests are two-sided.

1. The first six protective factors are derived from the Structured Assessment of Violence Risk in Youth (SAVRY). Protective factor items are scored as 0 = absent, 1 = present.
2. Participants self-reported the level of education completed by their (child-rearing) mother and father on a 1-9 scale: 1= no schooling, 2= some primary school, 3= completed primary school, 4= some high school, 5= completed high school, 6= some college, 7= completed college, 8= some graduate school, 9= completed graduate school.

Receiver Operating Characteristic Curves

Receiver Operating Characteristic (ROC) curves predicted conversion to psychosis among CHR participants (N = 283) who completed at least two MRI scans, with separate models employing PC_scan2_ and PC_Final_ in the left ROI as predictors as described in the main text. The following logistic regression models were tested for each left ROI PC value:

1. **PC Only:** Conversion (CHR-NC = 0, CHR-C=1) ~ PC
2. **PC + Scanner:** Conversion (CHR-NC = 0, CHR-C=1) ~ PC + scanner
3. **Full Model:** Conversion (CHR-NC = 0, CHR-C=1) ~ PC + age + age^2^ + sex + scanner.

Additionally, a model predicting conversion from only demographic predictors (age, age^2^, sex was compared to models including the PC term (same model for PC_scan2_ and PC_Final_). For each model, predicted outcomes (i.e. conversion vs. non-conversion) were compared to actual outcomes in an ROC curve using the *pROC* package [9] in R and each model’s performance was measured by calculating area under the curve (AUC; i.e. the model’s ability to discriminate between converters and non-converters). All model coefficients from the full model for PC_scan2_ and PC_Final_ are presented in **Table S14**. Additionally, coefficients for the PC term in the PC only and PC + scanner models are presented. Finally, antipsychotic medication was added as an additional model predictor in the full model and the medication effect for the PC_scan2_ and PC_Final_ are presented in the final row of the table. Including medication in the full model raised the AUC of the PC_scan2_ model to 0.75 and raised the AUC of the PC_Final_ model to 0.80.

| **Full Model** | **Z-value (PC_scan2_)** | **P-value (PC_scan2_)** | **Z-value (PC_Final_)** | **P-value (PC_Final_)** |
| --- | --- | --- | --- | --- |
| Intercept | -1.026 | 0.305 | -0.717 | 0.473 |
| Percent Change | -2.241 | 0.025 | -3.678 | <0.001 |
| Age | 0.255 | 0.799 | -0.118 | 0.906 |
| Age^2^ | -0.093 | 0.926 | 0.238 | 0.812 |
| Sex | -0.705 | 0.481 | -0.545 | 0.586 |
| Scanner 1 | -0.004 | 0.997 | -0.003 | 0.997 |
| Scanner 2 | 1.294 | 0.196 | 1.493 | 0.135 |
| Scanner 3 | -0.074 | 0.941 | 0.121 | 0.904 |
| Scanner 4 | -0.615 | 0.539 | -0.483 | 0.629 |
| Scanner 5a | -0.011 | 0.991 | -0.011 | 0.991 |
| Scanner 5b | -0.357 | 0.721 | 0.031 | 0.975 |
| Scanner 5c | 0.617 | 0.537 | 1.236 | 0.216 |
| Scanner 8a | -0.008 | 0.994 | -0.004 | 0.997 |
| Scanner 8b | 2.121 | 0.034 | 1.955 | 0.051 |
| Scanner 9 | 1.571 | 0.116 | 1.592 | 0.111 |
|  | | | | |
| **Model** | **Z-value (PC_scan2_)**  **PC Coefficient** | **P-value (PC_scan2_)**  **PC Coefficient** | **Z-value (PC_Final_)**  **PC Coefficient** | **P-value (PC_Final_) PC Coefficient** |
| PC Only | -2.37 | 0.018 | -3.983 | <0.001 |
| PC + Scanner | -2.27 | 0.023 | -3.738 | <0.001 |
| Full Model + Medication | -2.173 | 0.030 | -3.623 | <0.001 |
|  | | | | |
| Medication Coefficient | 1.073 | 0.283 | 1.923 | 0.054 |

**Table S14.** Coefficients for each term in the full logistic regression model (presented as Z-values) are provided for PC_scan2_ and PC_Final_ models in the left ROI, as well as the percent change term for all other models is provided. Adding antipsychotic medication as a covariate in the full logistic regression model does not change the significance of percent change effects. Across PC_scan2_ and PC_Final_ models, the percent change term in the left ROI is a significant predictor of conversion. In the PC_scan2_ model, the medication term is not significant and in the PC_Final_ model, there is a non-significant trend in which medication dosage is associated with conversion.

Pre-Conversion Only Results

The mean time to conversion for CHR-C was 8.8 months from first scan (SD = 7.6 months). A total of 9 scans in LME analyses were collected at or following time of conversion (4 scans in PC_scan2_ and PC_Final_ analyses). Excluding these scans in LME analyses at the cluster level and in PC_scan2_ and PC_Final_ analyses does not change the strength of significance of results (**Table S15)**.

|  | **Left ROI** | **Right ROI** |
| --- | --- | --- |
| **LME CHR-C vs. HC** | -4.455*** | -2.221* |
| **LME CHR-C vs. CHR-NC** | -2.851** | -0.875, ns |
| **PC_scan2_ CHR-C vs. HC** | -3.498** | -1.076, ns |
| **PC_scan2_ CHR-C vs. CHR-NC** | -2.118* | -0.128, ns |
| **PC_Final_ CHR-C vs. HC** | -5.243*** | -3.380*** |
| **PC_Final_ CHR-C vs. CHR-NC** | -4.397*** | -2.756** |

**Table S15.** T-statistics for LME, PC_scan2_, and PC_Final_ are not significantly altered when MRI scans collected at/following conversion are removed from analyses. P-value terms: ns > 0.05; * < 0.05; ** < 0.01; *** < 0.001.

**References:**

1. McGlashan T, Walsh B, Woods S. The psychosis-risk syndrome: handbook for diagnosis and follow-up. Oxford University Press; 2010.

2. Cannon TD, Sun F, McEwen SJ, Papademetris X, He G, van Erp TG, et al. Reliability of neuroanatomical measurements in a multisite longitudinal study of youth at risk for psychosis. Human Brain Mapping. 2014;35:2424–2434.

3. Takao H, Hayashi N, Ohtomo K. Effect of scanner in longitudinal studies of brain volume changes. Journal of Magnetic Resonance Imaging. 2011;34:438–444.

4. Liljequist D, Elfving B, Skavberg Roaldsen K. Intraclass correlation – A discussion and demonstration of basic features. PLoS One. 2019;14:e0219854.

5. Fusar-Poli P, Smieskova R, Kempton MJ, Ho BC, Andreasen NC, Borgwardt S. Progressive brain changes in schizophrenia related to antipsychotic treatment? A meta-analysis of longitudinal MRI studies. Neuroscience & Biobehavioral Reviews. 2013;37:1680–1691.

6. Navari S, Dazzan P. Do antipsychotic drugs affect brain structure? A systematic and critical review of MRI findings. Psychological Medicine. 2009;39:1763–1777.

7. Leucht S, Samara M, Heres S, Patel MX, Woods SW, Davis JM. Dose Equivalents for Second-Generation Antipsychotics: The Minimum Effective Dose Method. Schizophrenia Bulletin. 2014;40:314–326.

8. Cannon TD, Chung Y, He G, Sun D, Jacobson A, van Erp TGM, et al. Progressive Reduction in Cortical Thickness as Psychosis Develops: A Multisite Longitudinal Neuroimaging Study of Youth at Elevated Clinical Risk. Biol Psychiatry. 2015;77:147–157.

9. Robin X, Turck N, Hainard A, Tiberti N, Lisacek F, Sanchez J-C, et al. pROC: an open-source package for R and S+ to analyze and compare ROC curves. BMC Bioinformatics. 2011;12:1–8.
